# The genome sequence of the Brick, *Agrochola circellaris* (Hufnagel, 1766)

**DOI:** 10.12688/wellcomeopenres.18894.1

**Published:** 2023-01-31

**Authors:** Douglas Boyes, University of Oxford and Wytham Woods Genome Acquisition Lab, Darwin Tree of Life Barcoding collective, Wellcome Sanger Institute Tree of Life programme, Wellcome Sanger Institute Scientific Operations: DNA Pipelines collective, Tree of Life Core Informatics collective, Asia Hoile, Darwin Tree of Life Consortium

**Affiliations:** 1 UK Centre for Ecology and Hydrology, Wallingford, Oxfordshire, UK; 2 Department of Biology, University of Oxford, Oxford, UK

**Keywords:** Agrochola circellaris, the Brick, genome sequence, chromosomal, Lepidoptera

## Abstract

We present a genome assembly from an individual male *Agrochola circellaris* (the Brick; Arthropoda; Insecta; Lepidoptera; Noctuidae). The genome sequence is 572 megabases in span. Most of the assembly is scaffolded into 30 chromosomal pseudomolecules, including the Z sex chromosome. The mitochondrial genome has also been assembled and is 15.5 kilobases in length. Gene annotation of this assembly on Ensembl has identified 18,319 protein coding genes.

## Species taxonomy

Eukaryota; Metazoa; Ecdysozoa; Arthropoda; Hexapoda; Insecta; Pterygota; Neoptera; Endopterygota; Lepidoptera; Glossata; Ditrysia; Noctuoidea; Noctuidae; Xyleninae; *Agrochola*; *Agrochola circellaris* (Hufnagel, 1766)(NCBI:txid987866).

## Background

*Agrochola circellaris* (the Brick), is a species of moth belonging to the large and taxonomically controversial family Noctuidae within the superfamily Noctuoidea ( Regier *et al.*, 2017). The phylogeny of the Noctuoidae has been difficult to resolve, although considerable progress has been made using a sample of protein-coding genes ( Yang *et al.*, 2019).

*A. circellaris* is a relatively common moth in the UK, and is distributed throughout most of Europe, Asia Minor and Armenia. In the UK, the moth can be found in woodland, parkland and gardens, with the nocturnal adult on the wing between late August and early November ( Usher & Keiller, 1998). It can be identified by a distinctive dark spot inside the reniform stigma or ‘kidney mark’, with a diffuse band linking it to the leading edge of the wing. The forewings vary in colour between individuals, ranging from yellow to reddish brown ( Chinery, 1991); the genetic basis of the colour variation is unknown. The common name, ‘the Brick’, refers to the terracotta colour observed in many individuals.

The eggs overwinter and the larvae feed on flower heads and developing seeds of deciduous trees such as ash, poplar, sallow and wych elm. The larvae develop rapidly on this nutritious diet and, unusually, once fully developed they then spend several weeks inside a cocoon before pupation ( Butterfly Conservation, 2006). The species has been used in studies to assess the relation between the number of seeds damaged by *A. circellaris* caterpillars and stand density of field elm trees in Poland ( Bąk-Badowska *et al*., 2017). 

The genome of *A. circellaris* was sequenced as part of the Darwin Tree of Life Project, a collaborative effort to sequence all named eukaryotic species in the Atlantic Archipelago of Britain and Ireland. Here we present a chromosomally complete genome sequence for *A. circellaris*, based on one male specimen from Wytham Woods, Oxfordshire, UK.

## Genome sequence report

**Figure 1.**
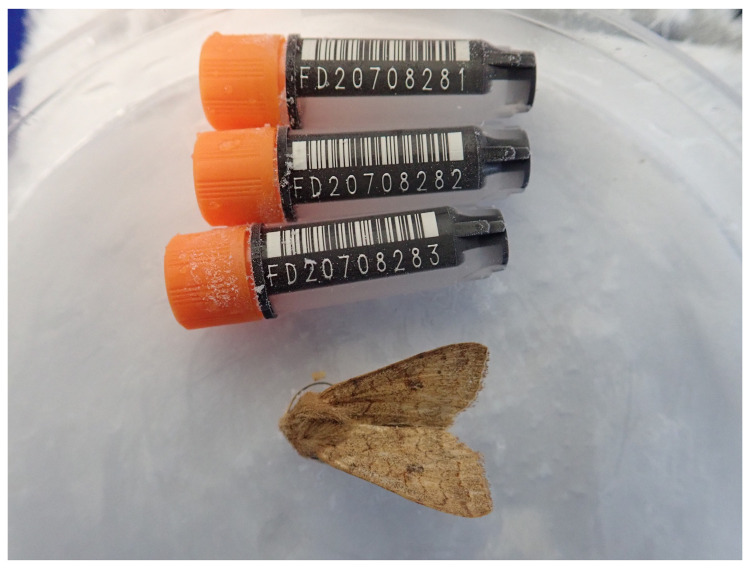
Photograph of the *Agrochola circellaris* (ilAgrCirc1) specimen used for genome sequencing.

The genome was sequenced from one male *Agrochola circellaris* ( Figure 1) collected from Wytham Woods (latitude 51.77, longitude –1.34). A total of 33-fold coverage in Pacific Biosciences single-molecule HiFi long reads and 73-fold coverage in 10X Genomics read clouds were generated. Primary assembly contigs were scaffolded with chromosome conformation Hi-C data. Manual assembly curation corrected four missing or mis-joins, reducing the scaffold number by 5.56%.

**Figure 2.**
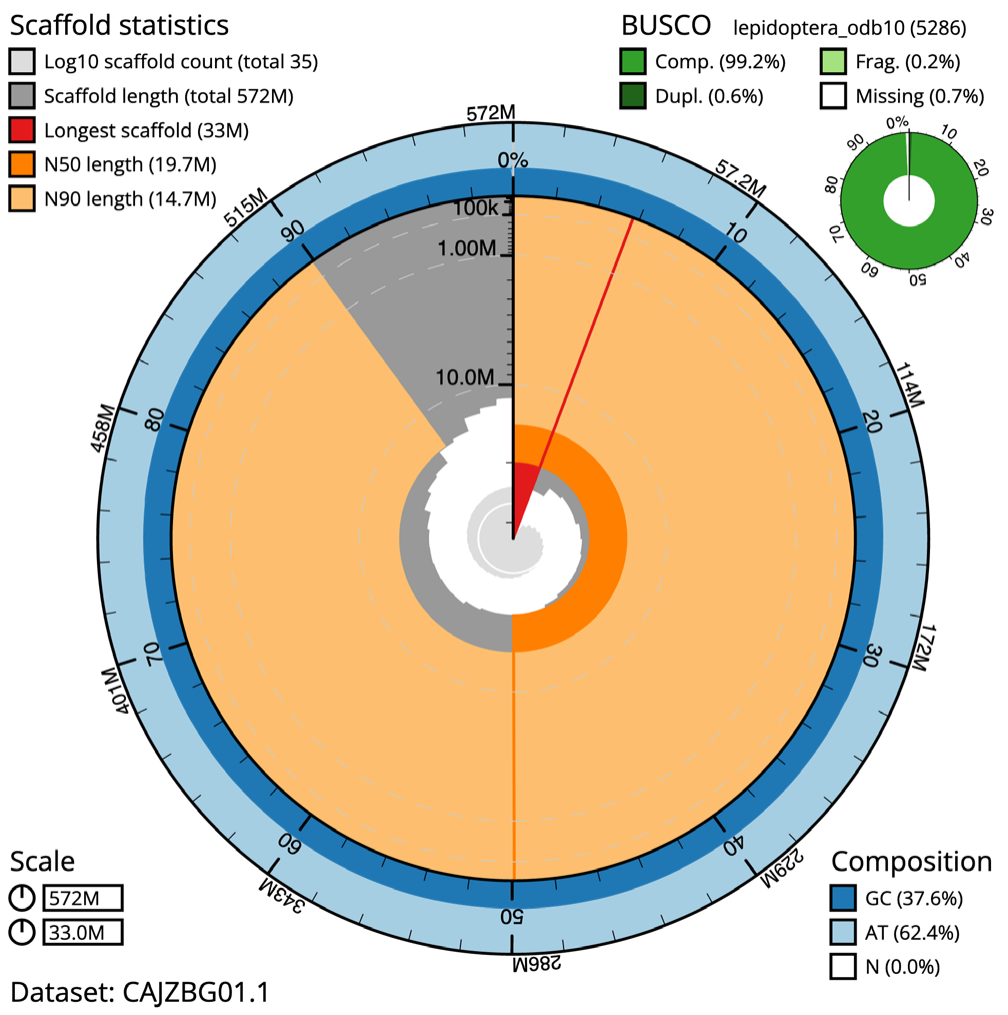
Genome assembly of *Agrochola circellaris*, ilAgrCirc1.1: metrics. The BlobToolKit Snailplot shows N50 metrics and BUSCO gene completeness. The main plot is divided into 1,000 size-ordered bins around the circumference with each bin representing 0.1% of the 572,162,832 bp assembly. The distribution of sequence lengths is shown in dark grey with the plot radius scaled to the longest sequence present in the assembly (32,976,333 bp, shown in red). Orange and pale-orange arcs show the N50 and N90 sequence lengths (19,711,079 and 14,656,473 bp), respectively. The pale grey spiral shows the cumulative sequence count on a log scale with white scale lines showing successive orders of magnitude. The blue and pale-blue area around the outside of the plot shows the distribution of GC, AT and N percentages in the same bins as the inner plot. A summary of complete, fragmented, duplicated and missing BUSCO genes in the lepidoptera_odb10 set is shown in the top right. An interactive version of this figure is available at https://blobtoolkit.genomehubs.org/view/ilAgrCirc1.1/dataset/CAJZBG01.1/snail.

The final assembly has a total length of 572.1 Mb in 34 sequence scaffolds with a scaffold N50 of 19.7 Mb ( Table 1). Most (99.99%) of the assembly sequence was assigned to 30 chromosomal-level scaffolds, representing 29 autosomes and the Z sex chromosome. Chromosome-scale scaffolds confirmed by the Hi-C data have been named in order of size ( Figure 2– Figure 5; Table 2). The assembly has a BUSCO v5.3.2 ( Manni *et al*., 2021) completeness of 99.2% (single 98.5%, duplicated 0.6%) using the lepidoptera_odb10 reference set ( *n* = 5,286). While not fully phased, the assembly deposited is of one haplotype. Contigs corresponding to the second haplotype have also been deposited.

**Table 1.** Genome data for *Agrochola circellaris*, ilAgrCirc1.1.

Project accession data		
Assembly identifier	ilAgrCirc1.1	
Species	*Agrochola circellaris*	
Specimen	ilAgrCirc1	
NCBI taxonomy ID	987866	
BioProject	PRJEB46316	
BioSample ID	SAMEA8603201	
Isolate information	male; ilAgrCirc1: thorax (PacBio and 10X sequencing); head (Hi-C) unknown sex; ilAgrCirc2: abdomen (RNA-Seq)	
Assembly metrics *		*Benchmark*
Consensus quality (QV)	59.5	*≥ 50*
*k*-mer completeness	100%	*≥ 95%*
BUSCO **	C:99.2%[S:98.5%,D:0.6%], F:0.2%,M:0.7%,n:5286	*C ≥ 95%*
Percentage of assembly mapped to chromosomes	99.99%	*≥ 95%*
Sex chromosomes	ZZ	*localised homologous pairs*
Organelles	Mitochondrial genome assembled	*complete single alleles*
Raw data accessions		
PacificBiosciences SEQUEL II	ERR6808001	
10X Genomics Illumina	ERR6688511–ERR6688514	
Hi-C Illumina	ERR6688510	
PolyA RNA-Seq Illumina	ERR9435003	
Genome assembly		
Assembly accession	GCA_914767755.1	
*Accession of alternate haplotype*	GCA_914767685.1	
Span (Mb)	572.1	
Number of contigs	39	
Contig N50 length (Mb)	19.7	
Number of scaffolds	34	
Scaffold N50 length (Mb)	19.7	
Longest scaffold (Mb)	33.0	
Genome annotation		
Number of protein-coding genes	18,319	
Number of gene transcripts	18,499	

* Assembly metric benchmarks are adapted from column VGP-2020 of “Table 1: Proposed standards and metrics for defining genome assembly quality” from ( Rhie *et al.*, 2021). ** BUSCO scores based on the lepidoptera_odb10 BUSCO set using v 5.3.2. C = complete [S = single copy, D = duplicated], F = fragmented, M = missing, n = number of orthologues in comparison. A full set of BUSCO scores is available at https://blobtoolkit.genomehubs.org/view/ilAgrCirc1.1/dataset/CAJZBG01.1/busco.

**Figure 3.**
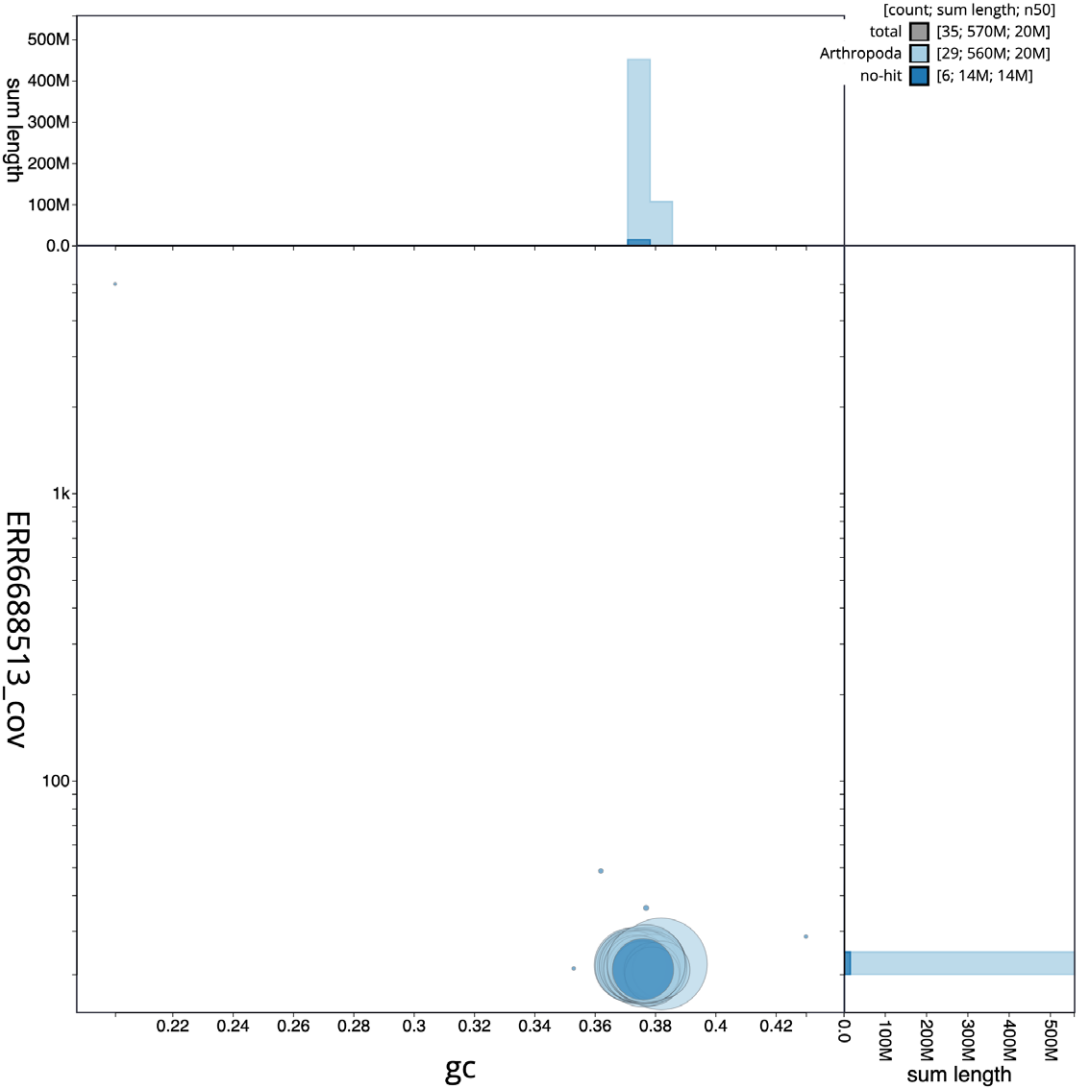
Genome assembly of *Agrochola circellaris*, ilAgrCirc1.1: GC coverage. BlobToolKit GC-coverage plot. Scaffolds are coloured by phylum. Circles are sized in proportion to scaffold length. Histograms show the distribution of scaffold length sum along each axis. An interactive version of this figure is available at https://blobtoolkit.genomehubs.org/view/ilAgrCirc1.1/dataset/CAJZBG01.1/blob.

**Figure 4.**
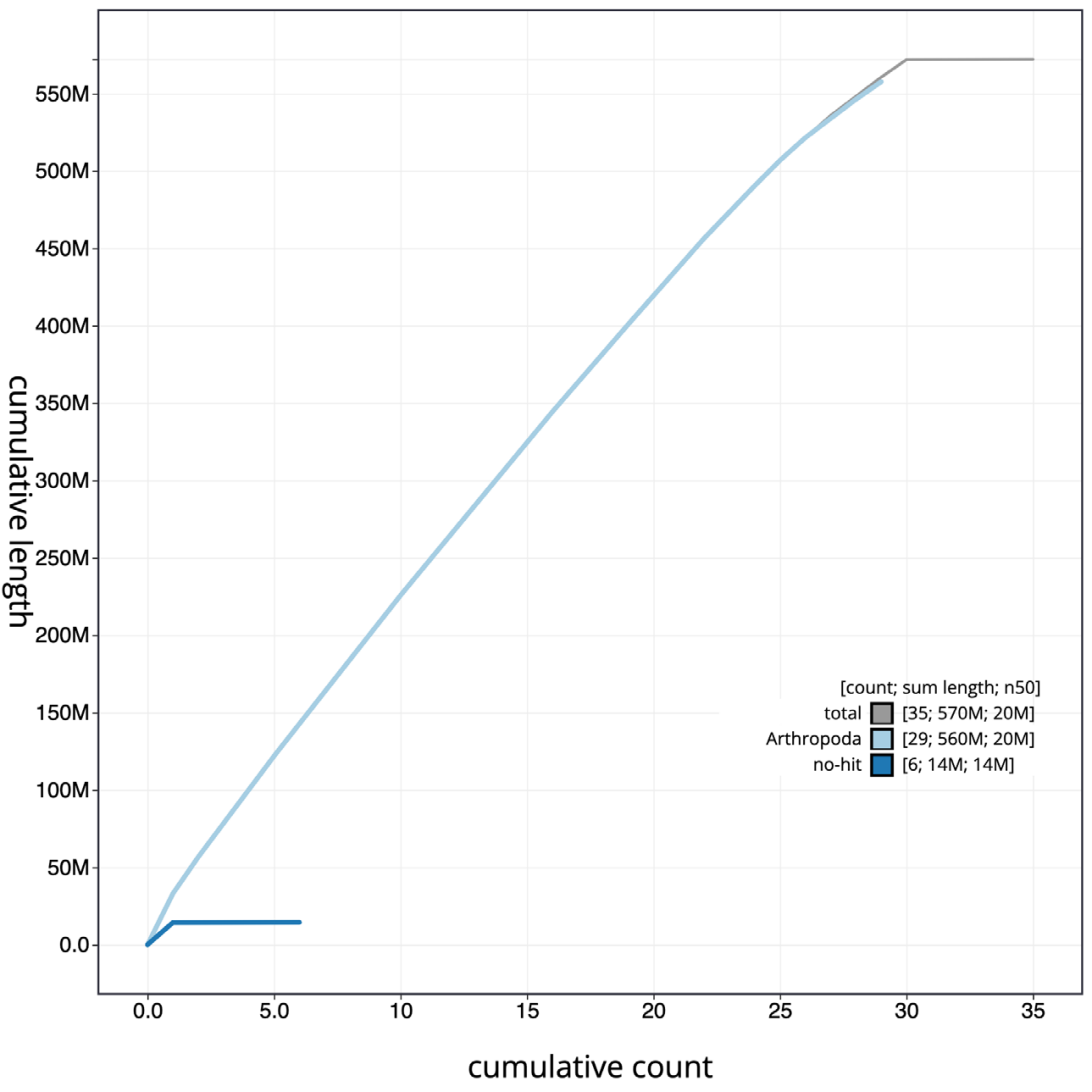
Genome assembly of *Agrochola circellaris*, ilAgrCirc1.1: cumulative sequence. BlobToolKit cumulative sequence plot. The grey line shows cumulative length for all scaffolds. Coloured lines show cumulative lengths of scaffolds assigned to each phylum using the buscogenes taxrule. An interactive version of this figure is available at https://blobtoolkit.genomehubs.org/view/ilAgrCirc1.1/dataset/CAJZBG01.1/cumulative.

**Figure 5.**
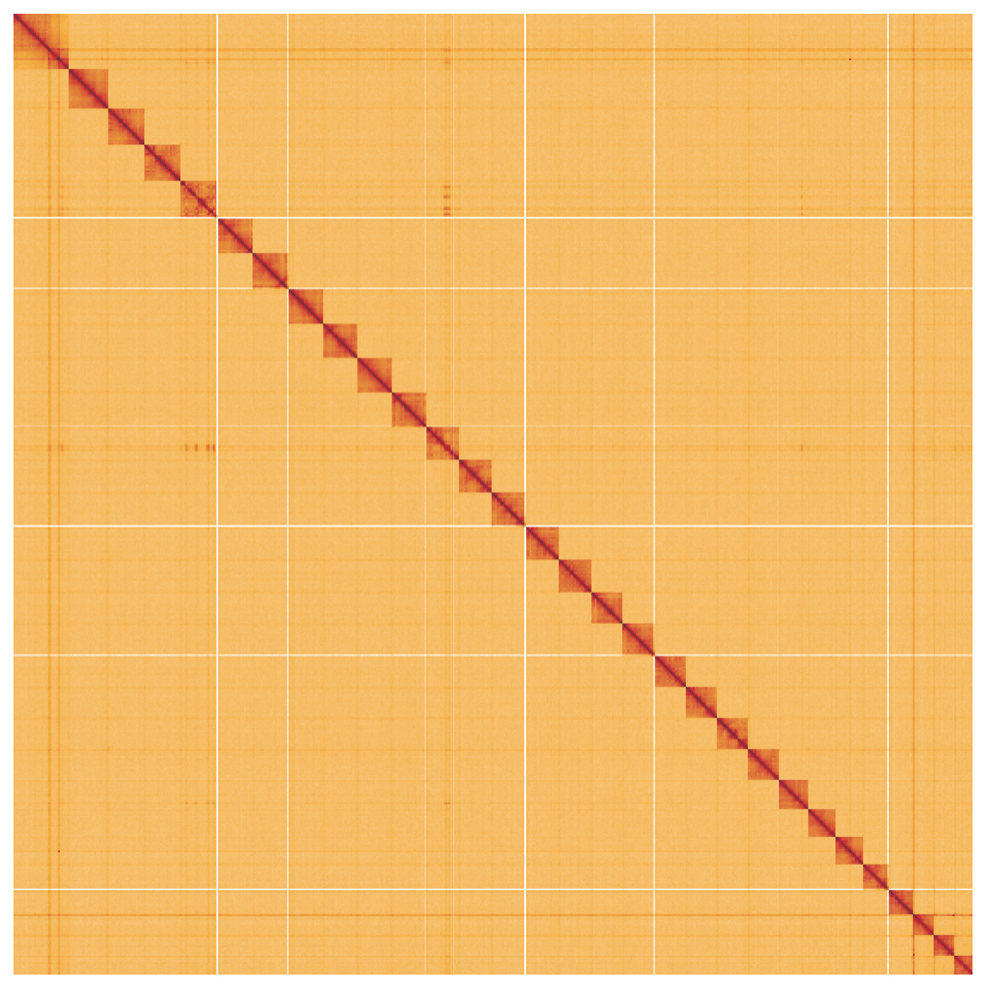
Genome assembly of *Agrochola circellaris*, ilAgrCirc1.1: Hi-C contact map. Hi-C contact map of the ilAgrCirc1.1 assembly, visualised using HiGlass. Chromosomes are shown in order of size from left to right and top to bottom. An interactive version of this figure may be viewed at https://genome-note-higlass.tol.sanger.ac.uk/l/?d=ZSISneTzRlK_n3QbgCsDxg.

**Table 2.** Chromosomal pseudomolecules in the genome assembly of *Agrochola circellaris*, ilAgrCirc1.

INSDC accession	Chromosome	Size (Mb)	GC%
OU611839.1	1	32.98	38.2
OU611841.1	2	22.02	37.5
OU611842.1	3	21.65	37.8
OU611843.1	4	21.65	37.6
OU611844.1	5	21.14	37.3
OU611845.1	6	20.91	37.2
OU611846.1	7	20.69	37.8
OU611847.1	8	20.61	37.3
OU611848.1	9	20.51	37.6
OU611849.1	10	20.18	37.7
OU611850.1	11	19.8	37.8
OU611851.1	12	19.76	37.6
OU611852.1	13	19.71	37.2
OU611853.1	14	19.69	37.4
OU611854.1	15	19.42	37.4
OU611855.1	16	18.94	37.5
OU611856.1	17	18.87	37.7
OU611857.1	18	18.78	37.9
OU611858.1	19	18.66	37.6
OU611859.1	20	18.61	37.3
OU611860.1	21	18.28	37.9
OU611861.1	22	17.31	37.4
OU611862.1	23	16.69	37.6
OU611863.1	24	16.39	37.5
OU611864.1	25	14.66	37.6
OU611865.1	26	14.3	37.6
OU611866.1	27	12.68	38.2
OU611867.1	28	12.1	37.9
OU611868.1	29	11.39	37.9
OU611840.1	Z	23.61	37.7
OU611869.1	MT	0.02	20.3
-	-	0.18	37.7

## Genome annotation report

The *A. circellaris* GCA_914767755.1 genome was annotated using the Ensembl rapid annotation pipeline ( Table 1; https://rapid.ensembl.org/Agrochola_circellaris_GCA_914767755.1/). The resulting annotation includes 18,499 transcribed mRNAs from 18,319 protein-coding genes.

## Methods

### Sample acquisition and nucleic acid extraction

Two *A. circellaris* specimens (ilAgrCirc1 and ilAgrCirc2) were collected in Wytham Woods, Oxfordshire (biological vice-county: Berkshire), UK (latitude 51.77, longitude –1.34) on 8 October 2020, using a light trap. The specimens were collected and identified by Douglas Boyes (University of Oxford) and snap-frozen on dry ice.

DNA was extracted at the Tree of Life laboratory, Wellcome Sanger Institute (WSI). The ilAgrCirc1 sample was weighed and dissected on dry ice with head tissue set aside for Hi-C sequencing. Thorax tissue was disrupted using a Nippi Powermasher fitted with a BioMasher pestle. High molecular weight (HMW) DNA was extracted using the Qiagen MagAttract HMW DNA extraction kit. Low molecular weight DNA was removed from a 20 ng aliquot of extracted DNA using 0.8X AMpure XP purification kit prior to 10X Chromium sequencing; a minimum of 50 ng DNA was submitted for 10X sequencing. HMW DNA was sheared into an average fragment size of 12–20 kb in a Megaruptor 3 system with speed setting 30. Sheared DNA was purified by solid-phase reversible immobilisation using AMPure PB beads with a 1.8X ratio of beads to sample to remove the shorter fragments and concentrate the DNA sample. The concentration of the sheared and purified DNA was assessed using a Nanodrop spectrophotometer and Qubit Fluorometer and Qubit dsDNA High Sensitivity Assay kit. Fragment size distribution was evaluated by running the sample on the FemtoPulse system.

RNA was extracted from abdomen tissue of ilAgrCirc2 in the Tree of Life Laboratory at the WSI using TRIzol, according to the manufacturer’s instructions. RNA was then eluted in 50 μl RNAse-free water and its concentration assessed using a Nanodrop spectrophotometer and Qubit Fluorometer using the Qubit RNA Broad-Range (BR) Assay kit. Analysis of the integrity of the RNA was done using Agilent RNA 6000 Pico Kit and Eukaryotic Total RNA assay.

### Sequencing

Pacific Biosciences HiFi circular consensus and 10X Genomics read cloud DNA sequencing libraries were constructed according to the manufacturers’ instructions. Poly(A) RNA-Seq libraries were constructed using the NEB Ultra II RNA Library Prep kit. DNA and RNA sequencing were performed by the Scientific Operations core at the WSI on Pacific Biosciences SEQUEL II (HiFi), Illumina HiSeq 4000 (RNA-Seq) and Illumina NovaSeq 6000 (10X) instruments. Hi-C data were also generated from head tissue of ilAgrCirc1 using the Arima v2 kit and sequenced on the Illumina NovaSeq 6000 instrument.

### Genome assembly

Assembly was carried out with Hifiasm ( Cheng *et al.*, 2021) and haplotypic duplication was identified and removed with purge_dups ( Guan *et al.*, 2020). One round of polishing was performed by aligning 10X Genomics read data to the assembly with Long Ranger ALIGN, calling variants with freebayes ( Garrison & Marth, 2012). The assembly was then scaffolded with Hi-C data ( Rao *et al.*, 2014) using SALSA2 ( Ghurye *et al.*, 2019). The assembly was checked for contamination as described previously ( Howe *et al.*, 2021). Manual curation was performed using HiGlass ( Kerpedjiev *et al.*, 2018) and Pretext ( Harry, 2022). The mitochondrial genome was assembled using MitoHiFi ( Uliano-Silva *et al.*, 2022), which performed annotation using MitoFinder ( Allio *et al.*, 2020). The genome was analysed and BUSCO scores generated within the BlobToolKit environment ( Challis *et al.*, 2020). Table 3 contains a list of all software tool versions used, where appropriate.

**Table 3.** Software tools and versions used.

Software tool	Version	Source
BlobToolKit	3.4.0	Challis *et al.*, 2020
freebayes	1.3.1-17- gaa2ace8	Garrison & Marth, 2012
Hifiasm	0.15.1	Cheng *et al.*, 2021
HiGlass	1.11.6	Kerpedjiev *et al.*, 2018
Long Ranger ALIGN	2.2.2	https://support.10xgenomics. com/genome-exome/ software/pipelines/latest/ advanced/other-pipelines
MitoHiFi	2	Uliano-Silva *et al.*, 2022
PretextView	0.2	Harry, 2022
purge_dups	1.2.3	Guan *et al.*, 2020
SALSA	2.2	Ghurye *et al.*, 2019

### Genome annotation

The BRAKER2 pipeline ( Brůna *et al.*, 2021) was used in the default protein mode to generate annotation for the *Agrochola circellaris* assembly (GCA_914767755.1) in Ensembl Rapid Release.

### Ethics/compliance issues

The materials that have contributed to this genome note have been supplied by a Darwin Tree of Life Partner. The submission of materials by a Darwin Tree of Life Partner is subject to the Darwin Tree of Life Project Sampling Code of Practice. By agreeing with and signing up to the Sampling Code of Practice, the Darwin Tree of Life Partner agrees they will meet the legal and ethical requirements and standards set out within this document in respect of all samples acquired for, and supplied to, the Darwin Tree of Life Project. Each transfer of samples is further undertaken according to a Research Collaboration Agreement or Material Transfer Agreement entered into by the Darwin Tree of Life Partner, Genome Research Limited (operating as the Wellcome Sanger Institute), and in some circumstances other Darwin Tree of Life collaborators.

## Data Availability

European Nucleotide Archive: *Agrochola circellaris* (the brick). Accession number PRJEB46316, https://identifiers.org/ena.embl/PRJEB46316 ( Wellcome Sanger Institute, 2021). The genome sequence is released openly for reuse. The *Agrochola circellaris* genome sequencing initiative is part of the Darwin Tree of Life (DToL) project. All raw sequence data and the assembly have been deposited in INSDC databases. Raw data and assembly accession identifiers are reported in Table 1.

## Author information

Members of the University of Oxford and Wytham Woods Genome Acquisition Lab are listed here: https://doi.org/10.5281/zenodo.4789928.

Members of the Darwin Tree of Life Barcoding collective are listed here: https://doi.org/10.5281/zenodo.4893703.

Members of the Wellcome Sanger Institute Tree of Life programme are listed here: https://doi.org/10.5281/zenodo.4783585.

Members of Wellcome Sanger Institute Scientific Operations: DNA Pipelines collective are listed here: https://doi.org/10.5281/zenodo.4790455.

Members of the Tree of Life Core Informatics collective are listed here: https://doi.org/10.5281/zenodo.5013541.

Members of the Darwin Tree of Life Consortium are listed here: https://doi.org/10.5281/zenodo.4783558.
