# Supplementary material for: The genome sequence of the Brick, Agrochola circellaris (Hufnagel, 1766)
Source: Wellcome Open Res. 2023 Jan 31;8:44. [Version 1] doi: 10.12688/wellcomeopenres.18894.1 (PMC12485328; doi:10.12688/wellcomeopenres.18894.1)
Supplement: Peer review history

# Reviewer report: Reviewer response for version 1

**DOI:** 10.21956/wellcomeopenres.20950.r132257

**Published:** 2025-09-30

**Authors:** Vahap Eldem

**Affiliations:** 1 Istanbul University, Istanbul, Turkey

I recommend acceptance of this data note following correction of one minor error. The manuscript successfully fulfills its intended purpose of documenting a high-quality genome assembly for Agrochola circellaris as part of the Darwin Tree of Life project. The assembly demonstrates excellent technical quality with a BUSCO completeness score of 99.2%, scaffold N50 of 19.7 Mb, and 99.99% of sequence assigned to chromosomal scaffolds. The methodological documentation is thorough and transparent, including all necessary software versions, protocols, and data accession numbers to ensure reproducibility. All raw sequencing data and assembled genomes have been properly deposited in public repositories with clearly reported accession identifiers.

 The scope of the manuscript is appropriate for a data note format. Rather than presenting novel biological insights or extensive comparative analyses, it focuses appropriately on documenting the generation and quality characteristics of a genomic resource that will be valuable to the research community. The assembly metrics meet or exceed the stated benchmarks adapted from current genome assembly standards. One correction is required before indexing: on page 3, line 4 of the Background section, "Noctuoidae" is incorrectly typed and should read "Noctuidae." This error was correctly identified by the previous reviewer. Additionally, while not essential for acceptance, the manuscript could be marginally strengthened by briefly mentioning expected heterozygosity levels given that an alternate haplotype assembly is referenced, and by explicitly noting that the absence of chromosome W is expected for the male ZZ specimen used. I have a minor methodological question regarding tissue selection. The study used thorax and head tissues from specimen ilAgrCirc1 for genomic sequencing and Hi-C respectively, while specimen ilAgrCirc2 contributed only abdominal tissue for RNA-Seq. Could the authors briefly clarify the rationale for this tissue selection strategy? I'm curious whether this was driven by practical constraints (e.g., tissue quality, specimen condition) or specific experimental considerations. While this doesn't affect the quality of the reported genome assembly, a brief explanation would be informative for readers planning similar projects.

Are sufficient details of methods and materials provided to allow replication by others?

Yes

Is the rationale for creating the dataset(s) clearly described?

Yes

Are the datasets clearly presented in a useable and accessible format?

Yes

Are the protocols appropriate and is the work technically sound?

Yes

Reviewer Expertise:

Mult,-omics of non-model organism

I confirm that I have read this submission and believe that I have an appropriate level of expertise to confirm that it is of an acceptable scientific standard.

# Reviewer report: Reviewer response for version 1

**DOI:** 10.21956/wellcomeopenres.20950.r72330

**Published:** 2024-02-12

**Authors:** Sivasankaran Kuppusamy

**Affiliations:** 1 Loyola College, Chennai, India

I'd like to appreciate the authors for putting together the whole genome sequence of *Agrochola circellaris*. The authors sequenced over 572 million base pairs for the genome assembly using 34 scaffolds. The authors found 18,499 gene transcripts and 18,319 protein-coding genes during genome assembly.

 Page no 3 second paragraph under the subheading “Background”. Line no 4. The phylogeny of the Noctuoidae……………..The family name Noctuidae was incorrectly typed. It should be Noctuidae not Noctuoidae. 

 Overall, the manuscript was well prepared.

Are sufficient details of methods and materials provided to allow replication by others?

Yes

Is the rationale for creating the dataset(s) clearly described?

Yes

Are the datasets clearly presented in a useable and accessible format?

Yes

Are the protocols appropriate and is the work technically sound?

Yes

Reviewer Expertise:

Molecular Entomology

I confirm that I have read this submission and believe that I have an appropriate level of expertise to confirm that it is of an acceptable scientific standard.
